# Sudden death caused by *Clostridium perfringens* sepsis presenting as massive intravascular hemolysis

**DOI:** 10.4322/acr.2020.185

**Published:** 2020-07-30

**Authors:** Katsuya Chinen

**Affiliations:** a Nerima General Hospital, Department of Pathology. Nerima, Tokyo, Japan.

**Keywords:** Clostridium perfringens, Sepsis, Hemolysis, Embolism, Fat, Death, Sudden

## Abstract

An 80-year-old Japanese woman with diabetes mellitus was admitted with gastrointestinal symptoms and pyrexia. At presentation, liver abscesses and severe hemolytic anemia were noted. Before detailed diagnostic evaluation and adequate treatment, she suddenly died 2.5 hours after admission. The autopsy and bacteriological examinations revealed liver abscesses and massive intravascular hemolysis caused by *Clostridium perfringens* as well as other miscellaneous critical pathological findings, including acute renal tubular necrosis, lung edema, and pulmonary fat embolism. In this article, the detailed autopsy results are described and clinicopathologic characteristics on *Clostridium perfringens-*related sudden death are discussed with a review of the literature.

## CASE REPORT

An 80-year-old Japanese woman was admitted with vomiting, diarrhea, and pyrexia, all of which had occurred acutely within 9 hours. She had no history of recent trauma. The patient was well until 9 hours prior to admission, when she presented with epigastralgia and vomiting. At midnight (3 hours prior to admission), her husband found that the patient had vomited in the bathroom and presented with incontinence of loose stool. At that time, she was febrile (axillary temperature 38.5 °C) with a normal level of consciousness. She was transferred to our hospital by ambulance early in the morning. Her past medical history included non-insulin dependent diabetes mellitus, hypertension, hyperlipidemia, uterine leiomyoma, and lumbar spinal canal stenosis. Her diabetes was well controlled and the patient did not have any diabetic complications. For the latter two conditions, hysterectomy and unilateral partial hemilaminectomy with bilateral ligamentectomy were performed 25 and 2 years earlier, respectively. She had no past history of hemolytic anemia.

**Figure 1:**
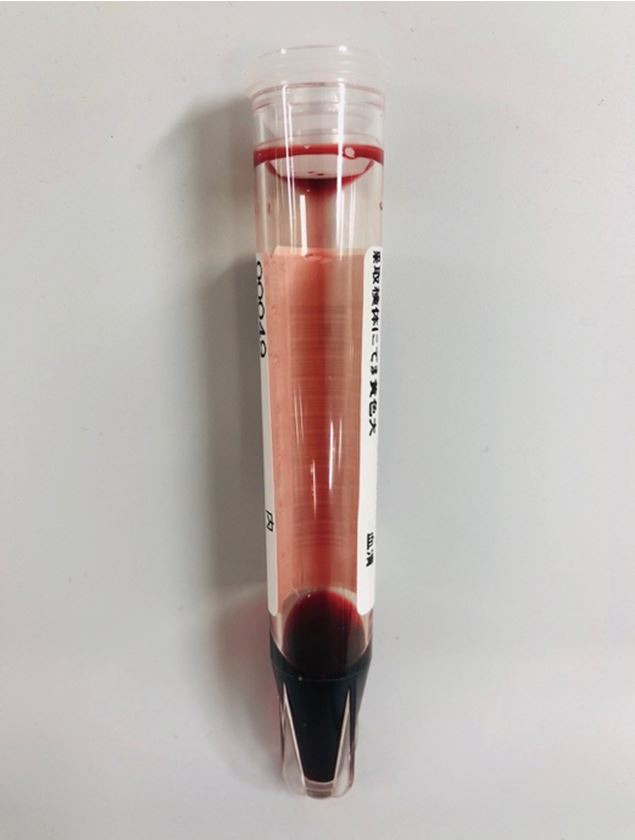
Serum of the patient at presentation.

**Figure 2:**
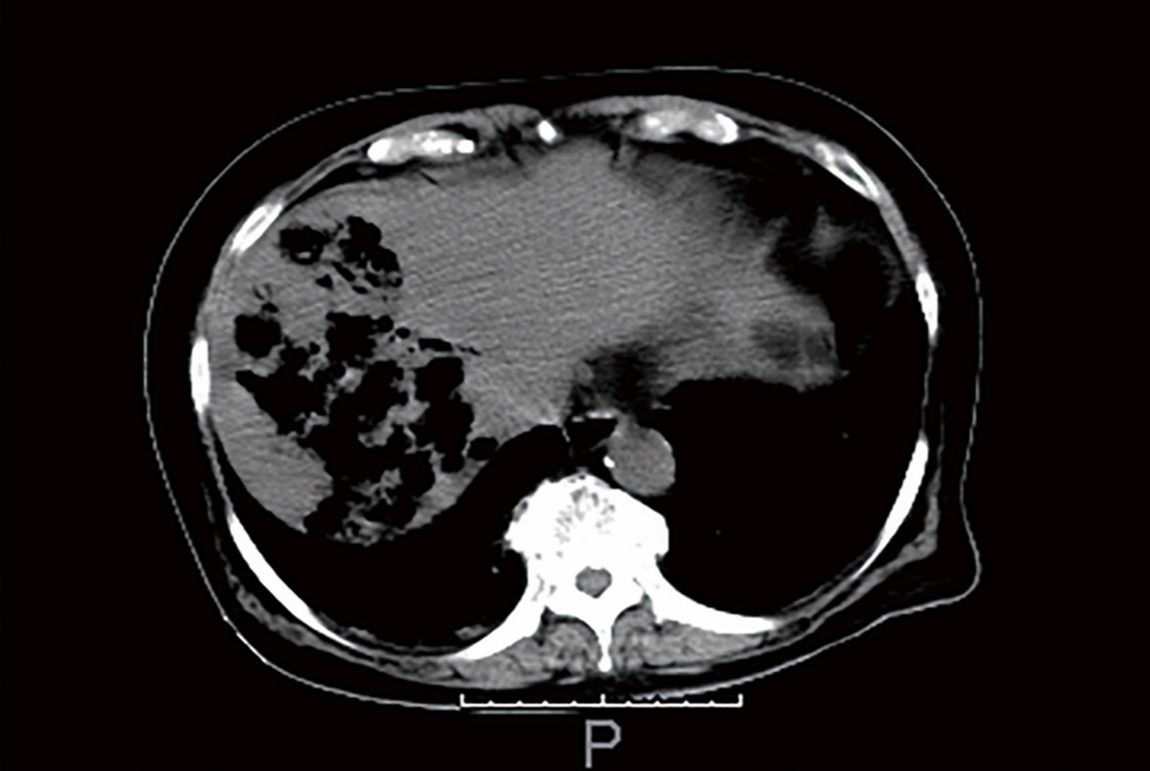
Non-contrast computed tomography of the abdomen. Irregular-shaped cavities containing abundant gas in the right lobe of the liver.

**Figure 3:**
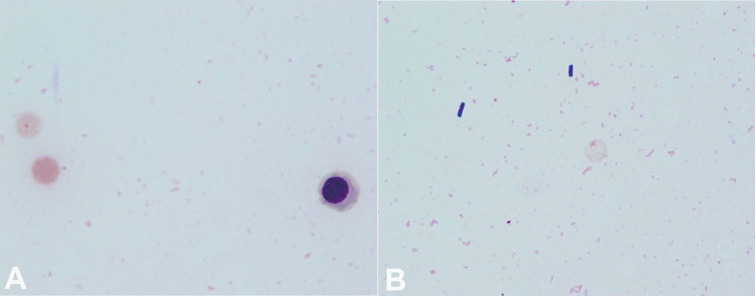
Microphotographs of the peripheral blood smear. **A –** A spherocyte showing loss of central pallor, a “dehemoglobinized” ghost cell, and an erythroblast are apparent. Note that intact red cells are no longer identified (Giemsa staining, 1000X); **B –** “Boxcar-shaped” bacilli are clearly demonstrated in the background of ghost cells and fragments of red cell membrane (Giemsa staining, 1000X).

At presentation, she was lethargic, but easily arousable. Initial vital signs were: blood pressure 127/48 mmHg; pulse 109 beats per minute; respiratory rate 21 breaths per minute; axillary temperature 38.4 °C; and oxygen saturation 92% on ambient air. Physical examination revealed a well-nourished, elderly woman who presented with pallor and mild jaundice. A systolic ejection murmur (Levine grade II/VI) was audible on the heart, and the lungs were clear to auscultation. Her abdomen, which showed a midline laparotomy scar, was distended and diffusely tender, but soft with no peritoneal signs. The extremities and skin were unremarkable. The patient’s peripheral blood was hemolyzed and the serum appeared exceptionally bright red, which was consistent with marked hemoglobinemia; that is, massive intravascular hemolysis (MIH) (Figure 1). The initial laboratory data measured by automated photometric assays are shown in Table 1. Anemia with non-physiologically reduced mean cell volume (MCV) and raised mean cell hemoglobin concentration (MCHC) was identified. No urine was obtained for urinalysis because the patient was anuric. Computed tomography (CT) of the abdomen revealed multiple gas-filled necrotic cavities in the right lobe of the liver (Figure 2) as well as distention of the small intestine. The former suggested liver abscesses. Soon after undergoing CT imaging, the patient presented with hypotension and bradycardia, rapidly deteriorated with agonal respirations and required orotracheal intubation and mechanical ventilation. Under the diagnosis of liver abscess and sepsis, fluid resuscitation was performed and intravenous treatment with 0.5 g of meropenem was started after taking two sets of blood cultures. Hypotension and bradycardia persisted in spite of adequate fluid resuscitation and increasing doses of catecholamine, and she eventually collapsed. Cardiopulmonary resuscitation (CPR) was unsuccessful and she died 2.5 hours after admission. After death, the peripheral blood smear that was prepared antemortem with Giemsa staining, revealed numerous spherocytes, “dehemoglobinized” ghost cells, debris of the red cell membrane, and a few erythroblasts; intact red cells were rarely identified (Figures 3A, 3B). There was no evidence of microangiopathy or parasitic infection indicating malaria or babesiosis. Occasionally, some “boxcar-shaped” bacilli were clearly identified in the same peripheral blood smear (Figure 3B). The blood cultures grew *Clostridium perfringens* (*C. perfringens*), *Escherichia coli* and Enterococcal species. The autopsy was carried out 5 hours after death.

**Table 1:** Laboratory data on admission

**Analyte**	**Result**	**NR**	**Analyte**	**Result**	**NR**
Leukocyte	3,600 /μL	(3,500-8,500)	AST	2,731 U/L	(10-35)
Erythrocyte	1,920,000/ μL	(3,700,000-4,900,000)	ALT	1,119 U/L	(5-40)
Hemoglobin	7.1 g/dL	(11.5-15.0)	LDH	10,975 U/L	(120-220)
Hematocrit	10.7%	(35-45)	Amylase	1,134 U/L	(31-107)
MCV	55.7 fL	(83-100)	Creatinine	1.94 mg/dL	(0.4-0.8)
MCH	37.0 pg	(28-34)	Urea	30 mg/dL	(8-20)
MCHC	66.4 g/dL	(32-36)	Glucose	250 mg/dL	(73-109)
Platelet	18,000 /μL	(150,000-350,000)	Sodium	138 mEq/L	(136-145)
TP	2.6 g/dL	(6.7-8.2)	Potassium	5.6 mEq/L	(3.6-4.8)
TB	3.55 mg/dL	(0.4-1.3)	Chloride	105 mEq/L	(99-107)
CK	31 U/L	(50-170)	CRP	2.25 mg/dL	(0-0.35)

ALT = alanine aminotransferase; AST = aspartate aminotransferase; CK = creatine kinase; CRP = C-reactive protein; LDH = lactate dehydrogenase; MCH = mean cell hemoglobin; MCHC = mean cell hemoglobin concentration; MCV = mean cell volume; NR = normal range; TB = total bilirubin; TP = total protein. Note: The hemolysis interferes with photometric assays (e.g. for TB, AST, and LDH) and their results are not necessarily valid.

## AUTOPSY FINDINGS

**Figure 4:**
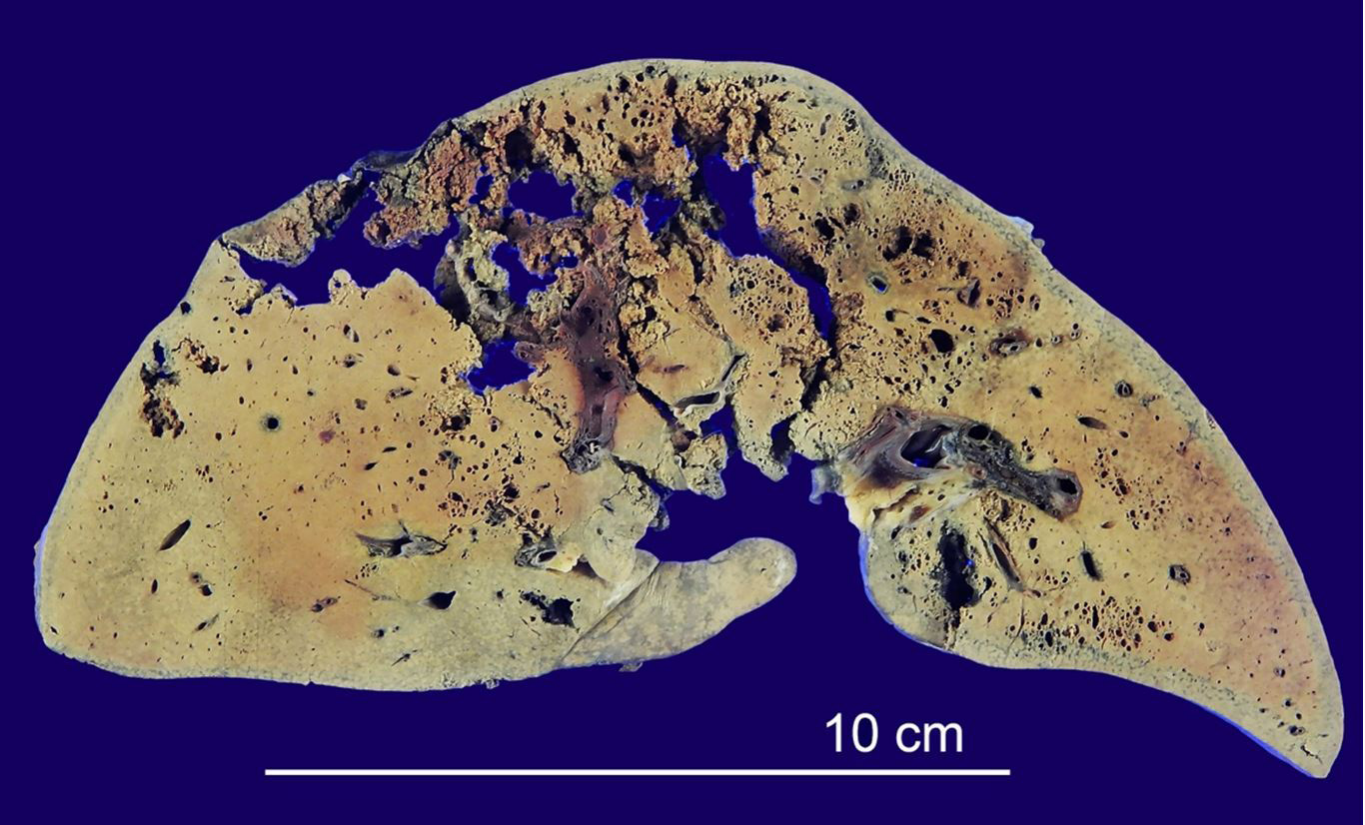
Abscesses featuring a honeycomb structure with gas bubbles, affecting the right and left lobes of the liver.

**Figure 5:**
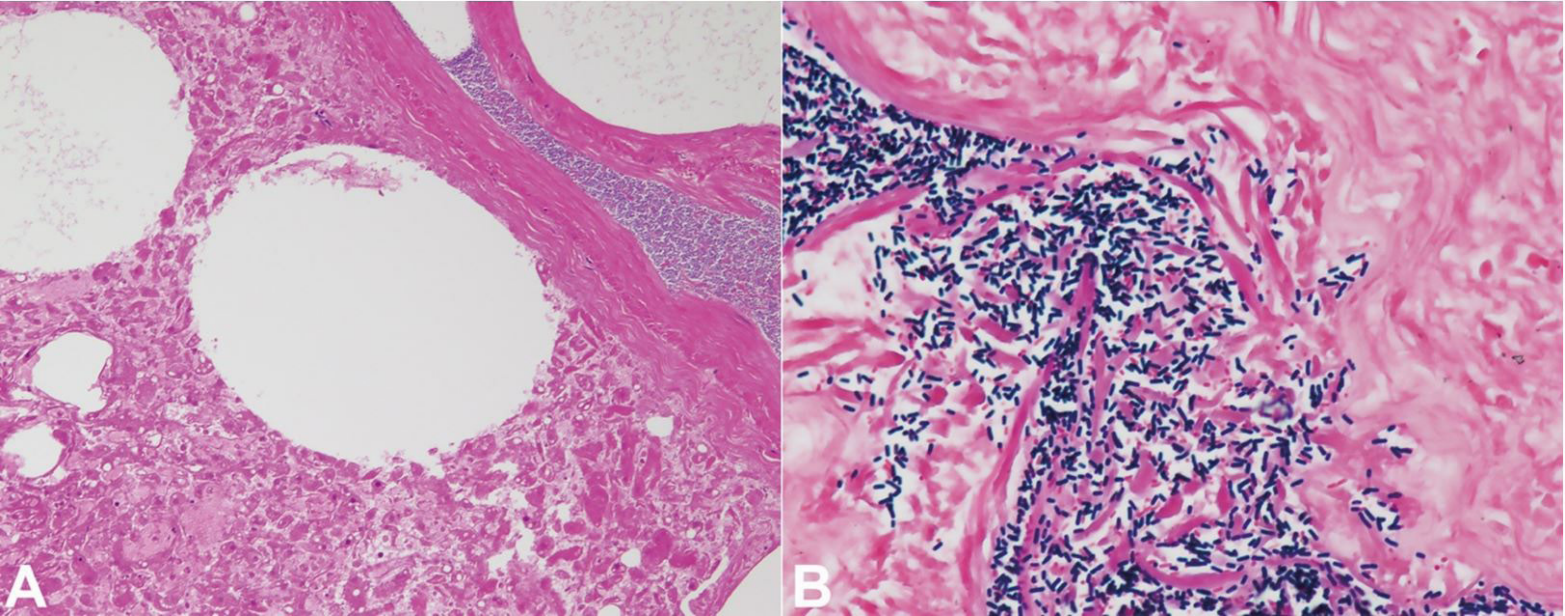
Microphotographs of the liver. **A –** Coagulation necrosis is evident in association with bleb formation. Bacterial proliferation is apparent in the right upper portion (H&E, 100X); **B –** Gram staining reveals marked proliferation of boxcar-shaped gram-positive bacilli. Note that inflammatory cell infiltration is lacking (400X).

**Figure 6:**
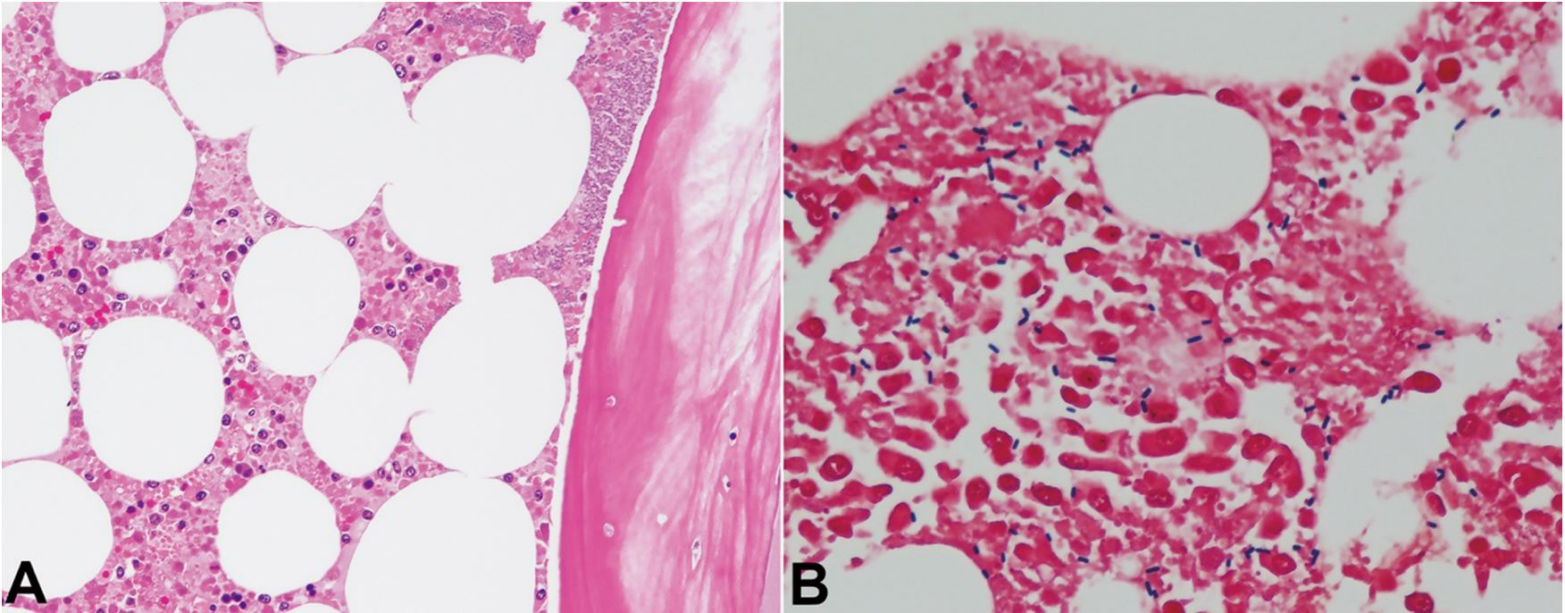
Microphotographs of the bone marrow. **A –** Necrosis of both hematopoietic cells and fat cells is apparent (H&E, 200X); **B –** Many boxcar-shaped gram-positive bacilli are confirmed (Gram staining, 400X).

**Figure 7:**
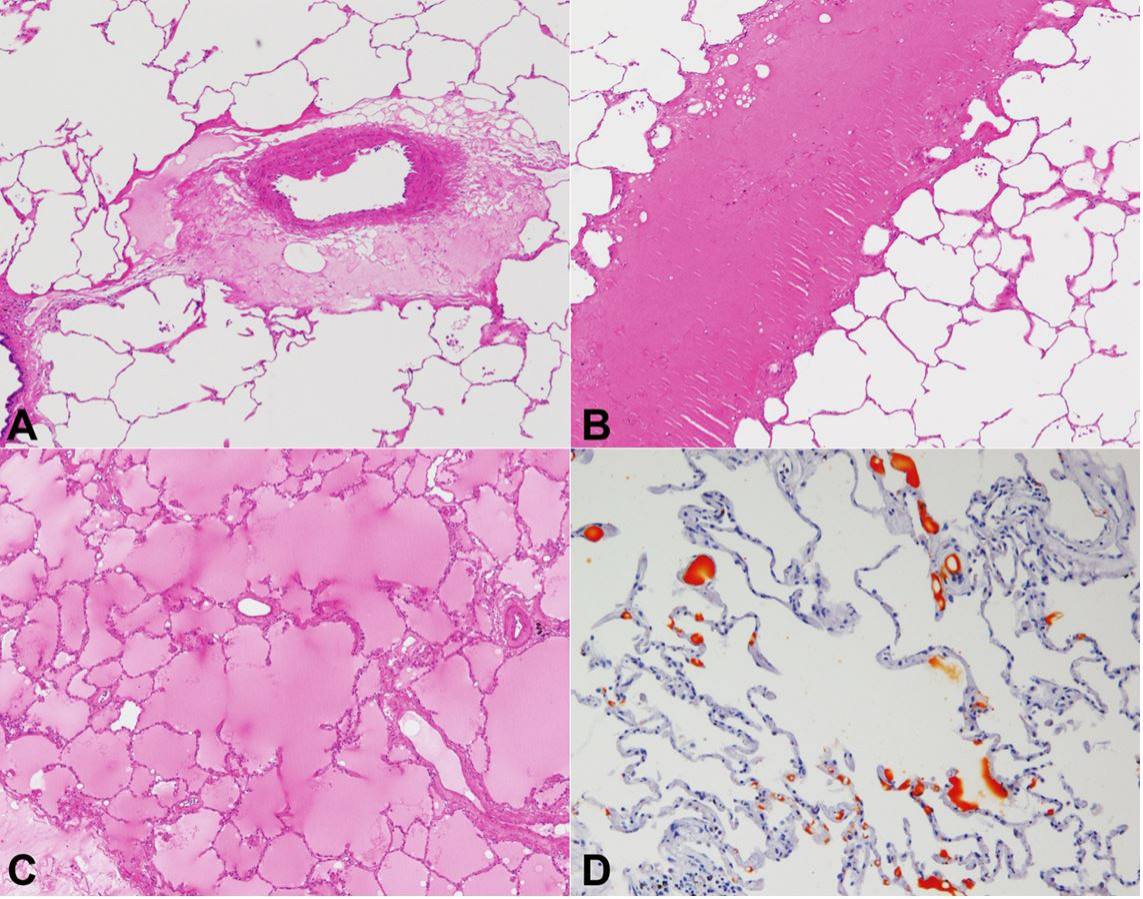
Microphotographs of the lung. **A –** Eosinophilic transudate around the pulmonary arteriole (H&E, 40X); **B –** Eosinophilic transudate is observed in the limited area within a pulmonary lobule (H&E, 40X); **C –** Area of panlobular edema (H&E, 40X); **D –** Fat globules are demonstrated within the pulmonary arterioles and capillaries of the interalveolar septa (Sudan III staining, 100X).

**Figure 8:**
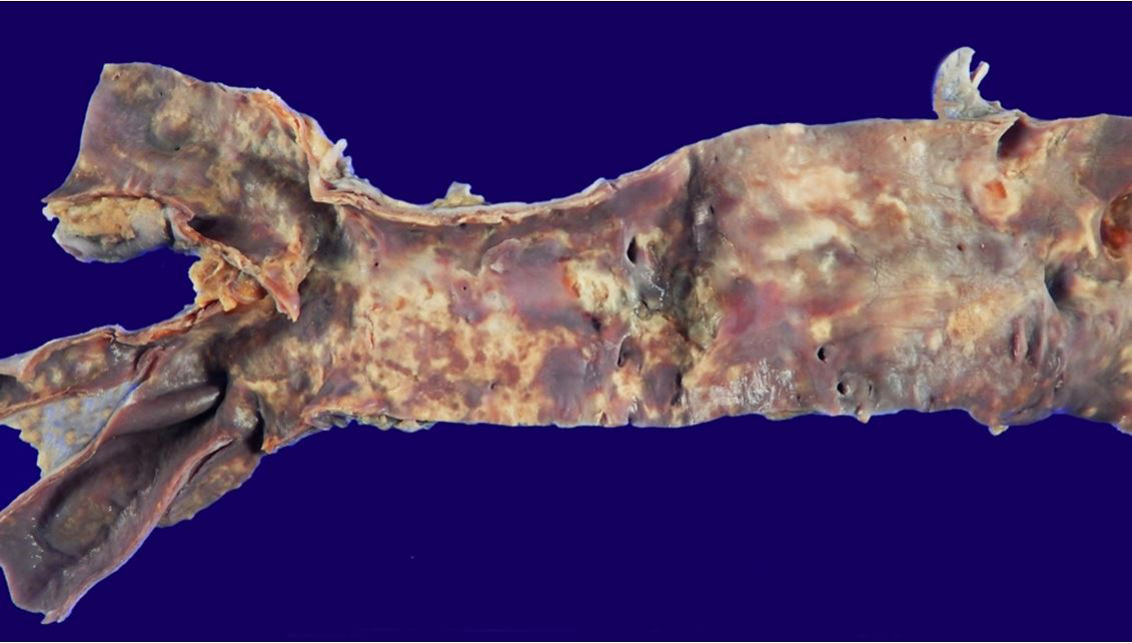
Aorta: moderate atherosclerosis; endothelium with a burgundy color, indicating massive intravascular hemolysis.

The liver, weighing 1,330 g (reference range [RR]: 345-1,250 g), was friable and smelled rotten. There were many emphysematous liver abscesses, up to 7 cm in long diameter, in both the right and left lobes (Figure 4). Microscopically, massive coagulation necrosis and gas bubble formation were evident with paucity of inflammatory cells (Figure 5A). The Gram staining revealed a large number of gram-positive bacilli, which showed a boxcar-shaped appearance (Figure 5B). Later, the bacilli were confirmed to be *C. perfringens* by a pus culture from the liver abscess. The spleen, weighing 80 g (RR: 70-95 g), was congestive and flabby. Microscopically, the red pulp was abundant in ghost cells in association with some clusters of boxcar-shaped gram-positive bacilli. In the bone marrow, necrosis of both hematopoietic cells and fat cells was evident in addition to many foci of boxcar-shaped gram-positive bacilli (Figures 6A, 6B). The pancreas showed scattered foci of necrosis with a few boxcar-shaped gram-positive bacilli. Most islets of Langerhans remained intact. The alimentary tract was unremarkable except for erosions of the small intestine, where the same bacteria were identified. The presence of the boxcar-shaped gram-positive bacilli was also confirmed in the adrenal glands and urinary bladder. Proliferation of gram-negative bacilli or gram-positive cocci was not observed anywhere. The left and right lungs, which weighed 650 g (RR: 85-500 g) and 740 g (RR: 100-620 g), respectively, showed scattered foci of lung edema. The edema varied in its degree, ranging from periarterial transudate to panlobular edema (Figures 7A, 7B, 7C). Interestingly, pulmonary fat embolism (PFE) was observed in the pulmonary arterioles and capillaries of the interalveolar septa, which was distributed diffusely in both lungs (Figure 7D). The kidneys (left 160g [RR: 50-150 g], right 175 g [RR: 40-150 g]) were dark red in color. Microscopic findings included extensive renal tubular necrosis with focal hemoglobin casts in tubular lumina. There was no evidence of disseminated intravascular coagulation (DIC), such as fibrin thrombus in the glomerular capillaries. The heart, weighing 470 g (RR: 150-480 g), was unremarkable except for left ventricular hypertrophy (20 mm thickness), with no finding of infarction or inflammation. The aorta showed moderate atherosclerosis and its endothelium was stained a burgundy color with hemoglobin (Figure 8). The latter was obvious pathological evidence of MIH.^1^ ^,^ ^2^

In summary, the autopsy, along with clinical, laboratory and microbiological data, revealed serious *C. perfringens* infection characterized by liver abscesses, sepsis, multiorgan bacterial infiltration, MIH, and miscellaneous critical lesions including acute renal tubular necrosis, lung edema, and PFE.

## DISCUSSION

*C. perfringens* is a gram-positive, spore-forming bacillus with boxcar-shaped morphology and is part of the commensal flora of the human digestive tract and female genital tract. It is also present in soil and inhabits in a wide range of species. Although generally classified as anaerobe, *C. perfringens* is somewhat “aero-tolerant”. Under optimal anaerobic conditions, it grows rapidly with a doubling time of about 7 minutes, accompanied by abundant gas production. It can become pathogenic.^3^ ^,^ ^4^ Historically, *C. perfringens* has been well known as a causative pathogen of gas gangrene (clostridial myonecrosis). In addition to gas gangrene, *C. perfringens* can cause heterogenous clinical manifestations, including gastroenteritis, liver abscess, cholecystitis, pancreatitis, central nervous system manifestations, endocarditis, sepsis, hemolysis, shock, and death.^5^ Its versatility as a pathogen is attributed to the production of a variety of toxins and virulent factors.^6^ ^,^ ^7^ *C. perfringens* produces at least 17 toxins, such as alpha-, theta-, epsilon-, beta-toxins, enterotoxin, neuraminidase, and hyaluronidase.^6^ ^-^ ^8^ Among them, alpha-toxin is the most significant.^5^ ^-^ ^9^ Alpha-toxin, a lecithinase called phospholipase C, hydrolyzes sphingomyelin and lecithin to phosphoryl choline and diglyceride; therefore, it can lyse red blood cells, platelets, endothelial cells, and the plasma membranes of muscle cells.^6^ ^,^ ^7^ In addition to the cytolytic nature, by its individual ability and/or synergistic effects with other toxins, alpha-toxin can be involved in many harmful reactions,^3^ ^,^ ^5^ ^-^ ^7^ including: (i) direct reduction in myocardial contractility; (ii) conduction defect in the heart; (iii) decreased mean arterial pressure; (iv) platelet aggregation and destruction; (v) microvascular injury; (vi) increased endothelial permeability; (vii) mistrafficking of neutrophils; (viii) hepatic mitochondrial dysfunction; (ix) erythrophagocytosis; and (x) stimulation of the production of endogenous mediators, such as tumor necrosis factor and platelet-activating factor. With these reactions, alpha-toxin can induce a variety of pathological processes, such as tissue necrosis, localized edema, electrolyte disturbance, intravascular coagulation, hemodynamic collapse, and multiorgan failure.

The patient suffered from serious *C. perfringens* infection, presenting as liver abscess, sepsis, and MIH, and these disorders were confirmed by the autopsy. The liver showed typical histological findings of a gangrenous liver abscess featuring coagulation necrosis, gas formation, proliferation of boxcar-shaped gram-positive bacilli, and paucity of inflammatory cells. In this case, the small intestine was assumed to be the most probable portal of entry, because the patient’s initial presentation was gastrointestinal symptoms, and there were erosions in the small intestine where gram-positive bacilli were identified. Also, the fact that *C. perfringens* sepsis was accompanied by bacteremia with other intestinal bacterial flora—*Escherichia coli* and enterococcal species—provides support for the hypothesis on the portal of entry for *C. perfringens* sepsis. Bacterial translocation of the bowel mucosa must have led to liver abscess by way of the portal system and then sepsis.

In the literature, like the present case, there are many MIH cases in which liver abscess was responsible for *C. perfringens* sepsis.^2^ ^,^ ^10^ ^-^ ^22^ As a cause of MIH, infections of the hepatobiliary system are more common than those of any other organs.^23^ ^-^ ^26^ This can be explained by the anatomical relationship between the hepatobiliary system and the digestive tract where *C. perfringens* inhabits as a commensal flora. The hepatobiliary system, especially the liver, is the most accessible organ for *C. perfringens* by way of both the portal vein and the bile ducts. Once *C. perfringens* infects the liver, it may grow rapidly with a more optimal anaerobic environment probably by the action of alpha-toxin with hepatic mitochondrial toxicity. Thereafter, owing to abundant blood flow, liver abscess may be prone to cause sepsis and then MIH.

In the case under discussion, in addition to the liver abscess, sepsis, and MIH, there were miscellaneous critical lesions, such as acute tubular necrosis of the kidneys, lung edema, and PFE. The acute tubular necrosis was considered to be attributed to severe hypoxia owing to MIH, hemodynamic collapse, and clostridial toxicity. The lung edema was relatively mild—ranging from periarterial transudate to panlobular edema—which was consistent with early-phase lung edema. Usually, the autopsy findings of lungs in patients dying of septic shock shows diffuse and massive lung edema (septic lung). The finding of lung edema limited to early phase suggests that the patient’s death was extremely sudden before massive lung edema could fully develop, indicating the fulminating process of *C. perfringens* sepsis. However, there may be some characteristic mechanisms for the development of lung edema peculiar to *C. perfringens* sepsis, probably toxin-related, which are different from mechanisms in other bacterial sepsis. The present case seems unique in that widespread PFE was demonstrated, since there have been no recent case reports indicating PFE as a complication of *C. perfringens* infection. In fact, the occurrence of widespread fat embolism in clostridial infections was clearly described in the 1940s by Govan.^27^ He reported three autopsy cases of *C. perfringens* infection in which fat embolism had been identified in the lungs, kidneys, brain, and spinal cord. The author also referred to the result of animal experiments in which injections of *C. perfringens* toxin had invariably given rise to PFE.^27^ It is likely that fat embolism may have been overlooked in recent autopsy studies for lack of specific fat stains. This may explain why fat embolism has not been noticed in patients with *C. perfringens* infection. In the present case, although the exact mechanisms for the development of PFE could not be determined, bone marrow necrosis, which is known to be an etiology of PFE,^28^ may have been responsible for PFE. Severe hypoxia as well as toxic attack due to *C. perfringens* sepsis must have caused bone marrow necrosis.^29^ In addition, hydrolysis of blood lipids as a result of phospholipase activity of the alpha-toxin may have been involved in the pathogenesis of PFE.^6^ ^,^ ^27^ On the other hand, it may be possible to consider PFE as a complication of sternal compression in CPR because PFE is frequently observed in the elderly subjected to such a procedure.^30^ Apart from CPR-related PFE, since PFE alone can be the cause of sudden death,^28^ it should be recognized as a possible fatal complication of *C. perfringens* infection.

From this case, we can learn a lesson; namely, *C. perfringens* infection is a dreadful disease that needs to be well-known to physicians and its pathophysiology is miscellaneous and complicated with multisystem organ failure. *C. perfringens* infection not only can cause a variety of critical conditions but also the catastrophic nature of the disease process sometimes may result in sudden death before any firm diagnosis is reached.^8^ ^,^ ^31^ In these circumstances, postmortem examinations have revealed an important pathological basis, and there are many case reports dealing with sudden death caused by *C. perfringens* infection. In order to better understand clinicopathologic characteristics of *C. perfringens*-related sudden death (CPRSD), a brief literature review was performed.

First, as shown in the present case, MIH is raised as the most important cause of CPRSD to be discussed.^9^ ^,^ ^15^ ^-^ ^22^ ^,^ ^32^ MIH occurs as a complication of *C. perfringens* sepsis and is frequently associated with severe anemia, acute renal failure, and DIC.^32^ MIH has classically been reported in cases of post-abortion and postpartum infections, and gas gangrene.^22^ ^,^ ^23^ ^,^ ^31^ ^,^ ^33^ ^,^ ^34^ Improvement in medical care has decreased the incidence of these entities.^22^ ^,^ ^34^ ^,^ ^35^ However, new and more aggressive treatment of patients with malignant diseases has increased the occurrence of serious infections, including clostridial sepsis.^22^ ^,^ ^36^ Currently, MIH develops in patients with diabetes mellitus, immunodeficiency, and malignant neoplasms, especially those with colon cancers and hematological malignancies.^1^ ^,^ ^4^ ^,^ ^7^ ^,^ ^10^ ^,^ ^23^ ^,^ ^37^ ^-^ ^40^ Impaired mucosal barrier of the intestine by malignant neoplasms and the toxic effect of chemotherapy tend to facilitate bacterial translocation and easy access to the bloodstream. Also, non-neoplastic diseases affecting the mucosa of the digestive system, such as pan-enteritis and intestinal arteriovenous malformation (vascular ectasia), can cause *C. perfringens*-induced MIH.^18^ ^,^ ^40^ In this context, surgical or interventional procedures on the hepatobiliary and/or gastrointestinal system—such as cholecystectomy, endoscopic retrograde cholangiopancreatography, and transhepatic arterial chemoembolization—also may be responsible for *C. perfringens*-induced MIH.^17^ ^,^ ^32^ ^,^ ^41^ ^,^ ^42^ Infrequently, the disease entity is reported to be caused by other conditions, such as emphysematous cystitis, endocarditis, degenerating uterine leiomyoma, and amniocentesis-related intrauterine infection.^29^ ^,^ ^43^ ^-^ ^45^ In some MIH cases, the potential source for *C. perfringens* sepsis remained unclear at autopsy.^1^ ^,^ ^46^

The *C. perfringens* alpha-toxin disrupts the fundamental integrity of the red blood cell membrane with its phospholipase activity.^9^ This mechanism is thought to result in development of spherocytes that are highly sensitive to osmotic lysis and subsequent hemolysis.^1^ ^,^ ^4^ During the hemolytic process, spherocytes, microcytes, ghost cells, and debris of the red cell membrane appear in the peripheral blood. These microscopic findings, which are pathognomonic for enzymatic toxin-related hemolysis, can be differentiated from those of mechanical red cell destruction (schistocytes), which are frequently observed as microangiopathy in DIC and hemolytic uremic syndrome.^11^ ^,^ ^20^ ^,^ ^24^ ^,^ ^32^

MIH progresses rapidly, shows high mortality (>70%), and death characteristically occurs within 24 hours of presentation as the result of cardiovascular collapse.^23^ ^,^ ^26^ ^,^ ^32^ ^,^ ^40^ Some MIH patients survived through a quick diagnosis and immediate treatment with the administration of an adequate dose of antibiotics, including penicillin G, prompt surgical procedure, and intensive care (e.g. exchange transfusion and hemodialysis).^11^ ^-^ ^14^ ^,^ ^23^ ^,^ ^25^ ^,^ ^40^ ^,^ ^41^ ^,^ ^44^ ^,^ ^45^ For the purpose of quick diagnosis, a high index of clinical suspicion is very important.^14^ ^,^ ^23^ ^,^ ^46^ Acute-onset and rapidly progressive hemolytic anemia is a key clinical manifestation. Laboratory data with both a non-physiological decrease in MCV and an increase in MCHC (meaning the presence of free hemoglobin and lysed red cells in the circulation) give clues for the diagnosis.^7^ ^,^ ^9^ ^,^ ^14^ ^,^ ^15^ ^,^ ^25^ In addition, direct visualization of spherocytes, ghost cells, and the debris of red cell membrane, with scant or a lack of intact red cells, can provide definite evidence for MIH.^20^ Furthermore, microscopic observation of boxcar-shaped gram-positive bacteria in a peripheral blood smear simultaneously may help to resolve the diagnostic dilemma for the etiology of MIH with confidence, because other causal infections, such as malaria and babesiosis, can be differentiated.^24^ There are some case reports in which the presence of gram-positive bacilli was demonstrated in the peripheral blood smear or buffy coat specimen, leading to a quick diagnosis of *C. perfringens* sepsis.^1^ ^,^ ^4^ ^,^ ^10^ ^,^ ^13^ ^,^ ^16^ ^,^ ^43^ In the present case, although Gram staining for the peripheral blood film was not performed, the presence of boxcar-shaped bacilli was clearly demonstrated in the peripheral blood smear that was routinely stained using the Giemsa method. This indicates that the peripheral blood smear can contribute immensely to the quick diagnosis of *C. perfringens*-induced MIH, provided that physicians and/or laboratory technicians appreciate the importance of the peripheral blood smear as an essential diagnostic tool.^20^ ^,^ ^32^

Apart from MIH, *C. perfringens* infection, with or without gas gangrene, can cause sudden death through a variety of pathophysiologic mechanisms, including acute renal failure, hyperkalemia, DIC, lung edema, pulmonary hemorrhage, and hemodynamic collapse (shock).^8^ ^,^ ^47^ ^-^ ^49^ As for primary infectious foci for CPRSD—in addition to the aforementioned lesions responsible for MIH—unusual causative lesions include acute tonsillitis, necrotizing enterocolitis, necrotizing pneumonia, and empyema.^47^ ^-^ ^49^ Although uncommon, once *C. perfringens* affects the cardiovascular system, the infection has an obvious risk for sudden death. Keese et al.^50^ reported a case of sudden cardiac death in which clostridial abscesses were identified in the myocardium. In case of clostridial aortitis, ruptured dissecting aneurysm can cause cardiac tamponade and sudden death.^51^ When *C. perfringens* affects splanchnic arteries, it may cause massive exsanguination and sudden death. Königsrainer et al.^52^ described a case of sudden death due to a ruptured hepatic artery caused by *C. perfringens* infection after pancreatic head resection. If abundant gas production occurs within the arteries of vital organs, it can cause circulatory disturbances and sudden death, as reported in the case of cerebral gas embolism.^53^ Moreover, some exceptional reported causes of CPRSD include meningitis after spinal surgery and secondary hemophagocytic syndrome in a pancreatic carcinoma patient.^54^ ^,^ ^55^ Considering the versatility of pathophysiology of *C. perfringens* infections, there must be a large spectrum of causes of CPRSD, presumably including examples that have not been reported so far in the literature. As presented in this article, *C. perfringens* sepsis-induced PFE may be just one example.

In practice, clostridial species other than *C. perfringens*, such as *Clostridium septicum* and *Clostridium sordellii* can also be responsible for sudden death.^8^ ^,^ ^56^ In addition, a wide range of animals are affected by clostridial infections and sometimes die suddenly.^57^ ^,^ ^58^ Considering that clostridial infection is one of zoonosis, understanding the clinicopathologic characteristics of clostridial infections in both humans and animals is very important.^59^ Viewed from a different angle, because the bacteria are present in soil, it is possible that clostridial infections may become epidemic when disasters, such as an earthquake, a tsunami, and terrorism occur and soil containing the bacteria is exposed to injured people.^56^ Therefore, from the viewpoint of emergency medicine, disaster medicine, veterinary medicine, and public health, physicians should be familiar with the pathophysiology and clinical details of clostridial infections as a fulminating and fatal disease.^5^ The accumulation of relevant cases and the analyses of pathophysiology are necessary for a greater understanding of CPRSD.

## CONCLUSION

The autopsy case of *C. perfringens* sepsis presenting with sudden death is reported herein. Since causes of CPRSD are miscellaneous and complicated, for better clinical understanding, the accumulation of studies on CPRSD is necessary from an interdisciplinary perspective.
